# 499. Level of Cord Blood Anti-SARs-CoV-2 Antibodies in Infants Born During 2021-2023 to The Mothers Who Had Received COVID-19 Vaccination During Pregnancy

**DOI:** 10.1093/ofid/ofad500.568

**Published:** 2023-11-27

**Authors:** Nophathai Sojisirikul, Keswadee Lapphra, Kulkanya Chokephaibulkit

**Affiliations:** Siriraj hospital/Mahidol University, Bangkok, Krung Thep, Thailand; Siriraj hospital/Mahidol university, Bangkoknoi, Krung Thep, Thailand; Siriraj hospital/Mahidol University, Bangkok, Krung Thep, Thailand

## Abstract

**Background:**

Maternal antibodies against SARS-CoV-2 actively transfer across placenta providing passive immunity to neonates. However, levels of cord blood anti-SARS-CoV2 antibodies and protective effects are still unclear. The objectives of the study was to investigate level of cord blood SARs-CoV-2 antibody of infants born to the mothers who received at least 1 dose of COVID-19 vaccine during pregnancy or within 3 months before pregnancy, as well as having SARS-CoV-2 infection during pregnancy.

**Figure:**
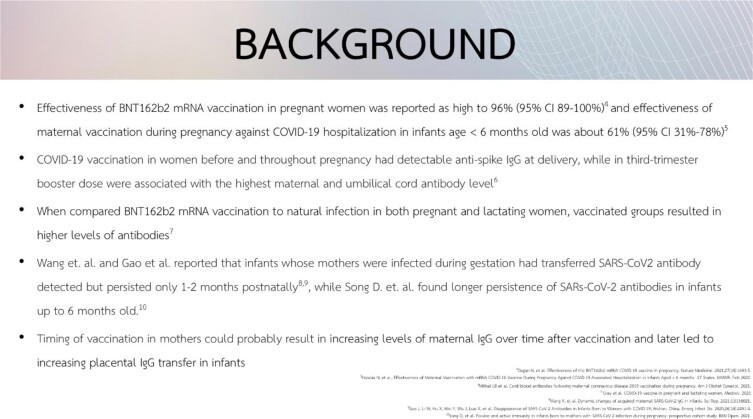

**Methods:**

Prospective observational study was conducted in Bangkok between March 2022 to March 2023. Cord blood samples were tested for anti-receptor binding protein IgG (anti-RBD IgG). Medical records were review for history of SARS-CoV-2 infections in the mothers.

**Figure:**
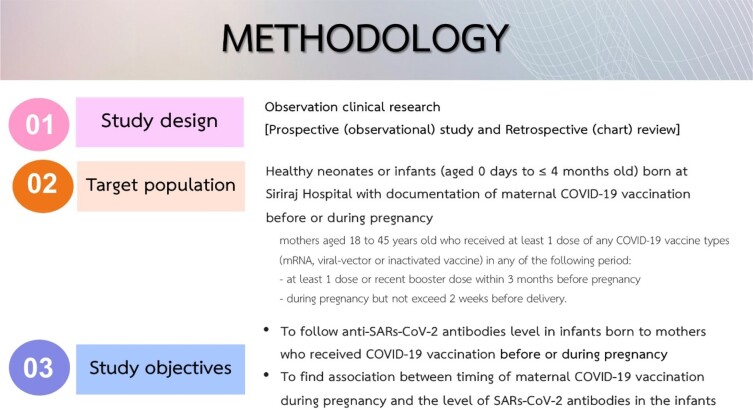

**Results:**

A total of 154 neonates were enrolled. The highest cord blood geometric mean concentration (GMC) of anti-RBD IgG was observed in infants who was born from the mother who received COVID-19 vaccine and were diagnosed with COVID-19 during pregnancy (n= 28; anti-RBD IgG: 7684.8 [95% confidential interval (CI) 4215.9-14007.8] AU/ml), while the lowest GMC was found in the infants born from mothers who received COVID-19 vaccination before pregnancy and did not have COVID-19 during pregnancy (n=18, anti-RBD IgG 1453.5 [95% CI 648.1-3259.6 AU/ml]. Maternal SARs-CoV-2 infection during pregnancy had a significant association with anti-RBD IgG levels (P=0.03).

**Figure:**
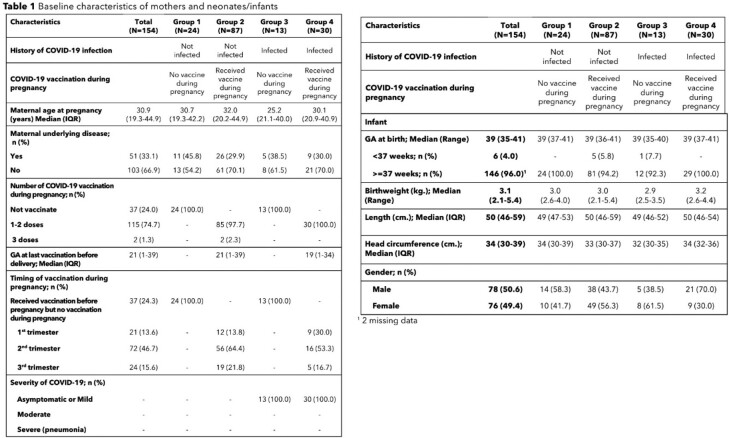

Figure of Anti-RBD IgG comparison at birth

**Figure:**
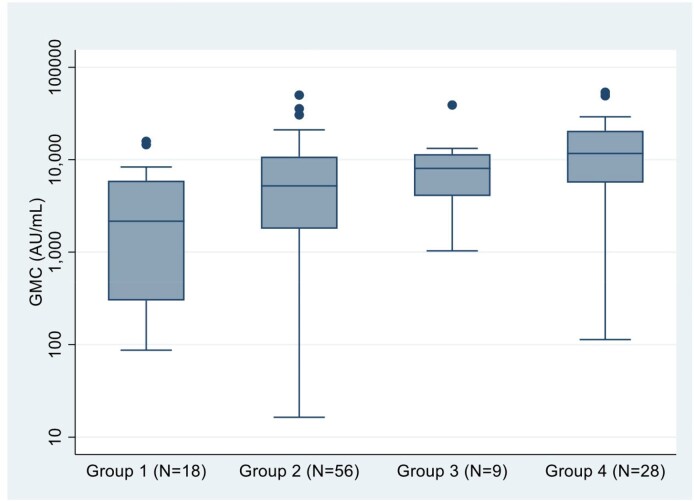

**Conclusion:**

Vaccinating and having SARs-CoV-2 infection during pregnancy induced several folds higher antibody level in cord blood that could effectively provide protection in infants.

**Figure:**
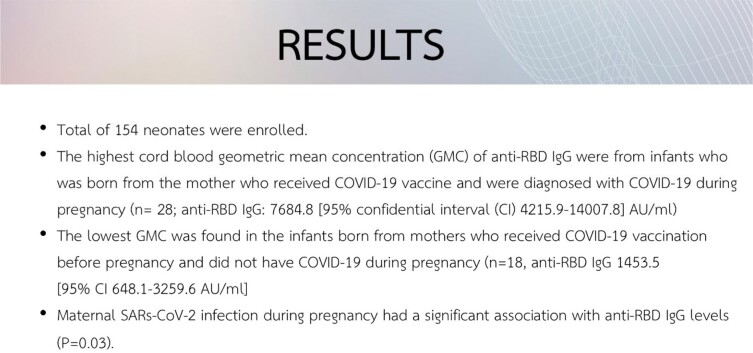

**Disclosures:**

**All Authors**: No reported disclosures

